# Symptom Locus and Symptom Origin Incongruity in Runner’s Dystonia – Case Study of an Elite Female Runner

**DOI:** 10.3389/fnhum.2021.809544

**Published:** 2021-12-16

**Authors:** Issei Ogasawara, Noriaki Hattori, Gajanan S. Revankar, Shoji Konda, Yuki Uno, Tomohito Nakano, Yuta Kajiyama, Hideki Mochizuki, Ken Nakata

**Affiliations:** ^1^Department of Health and Sport Sciences, Graduate School of Medicine, Osaka University, Osaka, Japan; ^2^Department of Rehabilitation, Faculty of Medicine, Academic Assembly, University of Toyama, Toyama, Japan; ^3^Institute for Transdisciplinary Graduate Degree Programs, Osaka University, Osaka, Japan; ^4^Department of Neurology, Graduate School of Medicine, Osaka University, Osaka, Japan; ^5^Department of Neurology, Sakai City Medical Center, Osaka, Japan

**Keywords:** movement disorder, task-specific focal dystonia, involuntary movement, motion capture system, electromyography, female athlete, yips, running

## Abstract

**Objectives:** Runner’s dystonia is a task-specific dystonia that occurs in the lower limbs and trunk, with diverse symptomatology. We aimed to identify the origin of a dystonic movement abnormality using combined three-dimensional kinematic analysis and electromyographic (EMG) assessment during treadmill running.

**Participant:** A 20-year-old female runner who complained of right-foot collision with the left-leg during right-leg swing-phase, which mimicked right-ankle focal dystonia.

**Results:** Kinematic and EMG assessment of her running motion was performed, which showed a significant drop of the left pelvis during right-leg stance-phase, and a simultaneous increase of right hip adductor muscle activity. This resulted in a pronounced adduction of the entire right lower limb with respect to the pelvis segment. Trajectories of right foot were seen to encroach upon left-leg area.

**Discussion:** These findings suggested that the symptom of this runner was most likely a form of segmental dystonia originating from an impaired control of hip and pelvis, rather than a distal focal ankle dystonia.

**Conclusion:** We conclude that, for individualized symptom assessment, deconstructing the symptom origin from its secondary compensatory movement is crucial for characterizing dystonia. Kinematic and EMG evaluation will therefore be a prerequisite to distinguish symptom origin from secondary compensatory movement.

## Introduction

Focal task-specific dystonia (FTSD) is a type of movement disorder that results in an abnormal involuntary muscle contraction of a focal body part during a specific well-learned task (Stahl and Frucht, 2017). FTSD has been frequently observed as writer’s cramps or musician’s dystonia in literature (Frucht, 2004; Goldman, 2015). One little-known phenomenon is runner’s dystonia (RD), symptoms characterized by an involuntary lower limb movement during running such as toe clawing/extension, ankle supination/inversion/eversion, and knee hyperextension (Leveille and Clement, 2008; Martino et al., 2009; Cutsforth-Gregory et al., 2016; Ahmad et al., 2018). When severe, the symptom also occurs during walking (Wu and Jankovic, 2006; McClinton and Heiderscheit, 2012; Cutsforth-Gregory et al., 2016). Foot and the lower limb muscles are commonly affected (Leveille and Clement, 2008), which may spread to the pelvis and trunk (Suzuki et al., 2011; Cutsforth-Gregory et al., 2016; Ahmad et al., 2018). Runners older than 40 years of age or those trained for a long time tend to suffer from this symptom (Schneider et al., 2006; Wu and Jankovic, 2006; Ramdhani and Frucht, 2013; Ahmad et al., 2018).

Routinely, surface electromyography (EMG) and X-ray/magnetic resonance imaging (MRI) of the lower limb are performed to identify abnormalities in the musculature or to explore a secondary basis including musculoskeletal problems (Schneider et al., 2006; McClinton and Heiderscheit, 2012; Ahmad et al., 2018). Most cases of RD are not associated with family history of movement disorders wherein genetic testing is performed to rule out DYT-1 phenotype dystonia (Schneider et al., 2006; Cutsforth-Gregory et al., 2016; Ahmad et al., 2018). Imaging of brain and spinal cord using MRI is frequently normal in most cases (Wu and Jankovic, 2006; McClinton and Heiderscheit, 2012). Visual inspections or offline video observation are often performed to investigate abnormal movement patterns (Schneider et al., 2006; McClinton and Heiderscheit, 2012). Very few studies have applied detailed motion capture assessment to quantify the joint kinematics associated with the RD symptoms (McClinton and Heiderscheit, 2012; Ahmad et al., 2018). Since RD is a rare pathology relative to the upper limb’s dystonia (Wu and Jankovic, 2006; Leveille and Clement, 2008; Martino et al., 2009) and the kinematic or muscle activity patterns are known to vary widely even among the small number of reported cases (Wu and Jankovic, 2006; Cutsforth-Gregory et al., 2016; Ahmad et al., 2018), the localization of symptomatic origin is therefore a diagnostic challenge.

Recently, a 20-year-old female elite runner presented to us with an abnormal, involuntary, right-ankle movement, consistently occurring during shoed running. Following examination by a general physician, she was diagnosed with RD of the right ankle and advised exercise-based physiotherapy for the right ankle. However, this physiotherapy intervention was unsuccessful. Since her problems persisted without relief, she was then referred to our department at Osaka University for a detailed evaluation of the problem.

To address and manage the athlete’s condition, we at Osaka University performed a dynamic evaluation of her running movement pattern using joint kinematics and surface EMG. Previous descriptive case studies have outlined movement pattern estimation mainly by visual inspection or by offline video observation (Schneider et al., 2006; Stan et al., 2020; Lee et al., 2021). However, subjective visual judgment of lower limb kinematics results in an inaccurate estimation of joint angles (Krosshaug et al., 2007), given that the resolution of visual observation is imprecise to identify the targeted motion of RD athletes. Therefore, a high-resolution objective measure such as the motion capture system combined with the dynamic surface EMG assessment was speculated to be ideal for an accurate quantification of the athlete’s spatiotemporal running patterns (Karp and Alter, 2017; Ahmad et al., 2018).

With respect to motion analysis, it is crucial to justify what an “abnormal” running pattern is. Given that movement patterns of RD patients are highly individualistic and stereotyped, we performed detailed, athlete-specific motion capture evaluation characterizing involved-uninvolved limb asymmetry that would define abnormal limb control. To that end, the aim of our study was to report the case of an athlete with lower limb task-specific dystonia to clarify spatiotemporal joint kinematics and dynamic surface EMG patterns of lower limb muscles to pinpoint symptom origin and explore whether the side-to-side limb asymmetry was localized only at the ankles. To define these changes within this athlete, we employed a sensitive statistical technique known as one-dimensional statistical parametric mapping (SPM) (Pataky et al., 2013) to explore the spatiotemporal asymmetries of truncal and lower limb kinematics and their associated EMG patterns during cyclic walking and running conditions. We hypothesized that the SPM comparison between affected and unaffected lower limbs as well as pelvic movement in running would reveal abnormal kinematic and EMG patterns to characterize the dystonic features in this runner.

## Method

### Ethical Considerations

This study was approved by the ethics review board of Osaka University Hospital (14250). Written informed consent was obtained from the athlete before data collection. Consent for publication was also obtained from this athlete.

### Patient Description

The patient was a 20-year-old female elite long-distance runner. Her first symptom appeared when she was around 18 years old. She gave a history that only during forward running, the medial side of her right forefoot collided with the medial aspect of the left calf during the right-leg swing-phase (see Supplementary Video). She was able to walk forward, backward, and sideways normally. Brain, spinal cord, and lower-limb MRI were characteristically normal. The cerebrospinal fluid examination also showed no findings to suggest any phenotypes of genetic dystonia. She had a normal motor development in her childhood. No family histories were identified for any movement disorders.

### Preparation for Motion Analysis

The motion-capture analysis for the athlete was performed at Osaka University 2 years and 4 months after the first diagnosis of RD in ankle elsewhere. The athlete wore a black-colored spandex shirt and pants with her own running shoes (Tarther Japan Black 1013A007, ASICS, Japan). Forty reflective markers were attached to the body landmarks (Table 1) and four marker-cluster plates with three reflective makers on each were placed on both side thigh and shank segments for the optical motion analysis. After skin preparation, the wireless surface EMG sensors (Trigno Avanti system, Delsys, Inc., United States) were fixed to vastus medialis (VM), semitendinosus (ST), gluteus medius (GM), hip adductor longus (HAL), tibialis anterior (TA), and lateral head of gastrocnemius (GC) of both legs. The sensors were firmly covered with the elastic tape to minimize movement artifacts. To protect the damage of foot collision, the posterior aspect of left calf was covered with the elastic tape. The athlete wore safety harness to prevent falling. We ascertained that the harness did not impede her locomotion. The static posture trial was captured with full maker set calibration, and then some markers (see Table 1) were removed before treadmill trials.

**TABLE 1:** Maker name and position.

Marker Name	Side	Position	Remove
TOE	Both	Anterior tip of shoe sole, 1 cm above from shoe sole surface.	
MMP	Both	Aiming at the head of first metatarsal bone on the shoe.	*
LMP	Both	Aiming at the head of fifth metatarsal bone on the shoe.	
FBC	Both	Aiming at base of third metatarsal bone on the shoe.	
CAL	Both	Most posterior edge of shoe heel wedge, 1 cm above shoe sole surface.	
MAKL	Both	Most prominent point of medial malleolus.	*
LAKL	Both	Most prominent point of lateral malleolus.	
MKNEE	Both	Most prominent point of medial femoral epicondyle.	*
LKNEE	Both	Most prominent point of lateral femoral epicondyle.	
TTB	Both	On the mid of tibial tuberosity.	
ATH	Both	Anterior aspect of thigh segment, approximately mid-way of hip and knee joint.	
GT	Both	Most laterally prominent point of great trochanter.	
ASIS	Both	Most prominent point of anterior superior iliac spine.	
PSIS	Both	Most prominent point of posterior superior iliac spine.	
SCRM	Center	On the mid of sacrum.	
STRN	Center	On the top edge of sternum.	
C7	Center	Most prominent point of seventh cervical spinous process.	
SHD	Both	Most prominent point of acromion process.	
ELB	Both	Most prominent point of the lateral humeral epicondyle.	
WRIST	Both	Most prominent point of the ulnar styloid process.	
HND	Both	On the head of third metacarpal bone.	
HEAD	Center	Tip of head.	

Remove * – Markers were removed after static calibration trial since those markers were potentially problematic due to foot collision symptom. The position of the removed marker was reconstructed by information of marker clusters or other markers on the same segment.

### Treadmill Walking and Running Trial

The athlete was asked to perform a walking to running task on the electric treadmill (MYRUN model: DCKN1B, Technogym S.p.A, Italy). A total of six trials were performed. Each trial lasted approx 2.5 min long. The athlete initially took a static pose on the treadmill, then gradually increased the speed of the treadmill to 6.0 km/h by herself, and performed fast walking for about 50 steps. When cued by the experimenter, the athlete started to run at the same speed and performed another 60 running steps. The running speed of 6.0 km/h was the lowest speed to induce her symptom. To measure the athlete’s natural performance, no specific instructions were given on how to walk and run. The athlete was allowed to stop running at any time she felt sustained running would be injurious.

### Data Collection

The 3D positions of the body markers were captured with the 12 optical cameras (OptiTrack Prime 17W, Software: Motive version 1.9, NaturalPoint, Inc., United States) with a sampling frequency of 360 Hz. The EMG signals from the selected muscles were sampled at 2,000 Hz with the Delsys Trigno Avanti sensors and measured using LabChart version 8.0.9 (ADInstruments, United States). A clock device (eSync2, NaturalPoint, Inc., United States) was used to synchronize the OptiTrack and LabChart. For offline visual inspection, video recordings from the rear and on the right side of the athlete were taped (HDR-PJ800, 30 fps, SONY, Japan). The EMG signal during the maximum voluntary contraction (MVC) test (two repetitions of 2 s MVC for each muscle with intensive verbal encouragement) was collected for offline signal normalization.

### Data Analysis and Assessment Variables

Offline data analysis was performed with custom scripts written in Scilab 6.01 (ESI Group, France). The motion capture data were smoothed with the second-order Butterworth digital filter (low-pass, zero-lag, cutoff-frequency of 10 Hz). Since the athlete was a typical heel-first contact runner, the timing of heel contact (HC) was identified as the local minimums observed in the vertical component of the heel marker “CAL.” The timing of toe-off (TO) was judged when the first increase of vertical component of the toe-marker “TOE” appeared after HC. One gait cycle was defined as the period from the previous HC to the next HC for each leg. Data for one gait cycle was normalized to 101 data points (0–100%). One gait cycle was consisted of the stance-phase (HC to TO) followed by the swing-phase (TO to the next HC).

The seven-link kinematic model, consisting of both feet, shanks, thighs, and one pelvis segment, was constructed using the time-normalized marker data. The local coordinate system was defined for each segment. For the kinematic assessment of athlete movement, hip adduction(+)/abduction(−), hip flexion(+)/extension(−), hip internal(+)/external(−) rotation, knee flexion(+)/extension(−), ankle adduction(+)/abduction(−), and ankle dorsi(+)/planter(−) flexion were calculated as time-series kinematic variables. To evaluate the contralateral pelvis-drop at the stance-phase, we calculated the local minimum of the vertical component of both-side ASIS markers during one gait cycle (the lowest value occurred in one gait cycle) was determined and was offset with the static trial. To visualize the three-dimensional (3D) foot trajectory relative to the pelvis segment, the position vector going from the center of pelvis segment (mid-point of two ASIS and two PSIS markers) to the center of foot segment (FBC marker) was calculated and expressed with the pelvis coordinate system. To quantify the severity of right-foot collision to the left calf, the distance from the right-foot’s FBC marker and the left shank segment (e.g., foot-calf distance) was calculated based on the measured marker data. The simulated foot-calf distance was also calculated assuming that the right-ankle position was maintained appropriately with respect to the left-ankle position (assuming that there was no side-to-side difference in the ankle position).

Electromyographic signals during trials were high-pass filtered (5 Hz), full-wave rectified, and low-pass filtered (10 Hz) with a second-order zero-lag Butterworth digital filter to obtain enveloped signals. The same procedure was applied to the MVC trials, and the peak MVC value was detected for each muscle. EMG signals during trials were normalized to the peak MVC values (%MVC). Single gait/running cycle EMG data were also time normalized to 101 data points synchronizing with motion capture data.

### Statistical Analysis

To assess the side-to-side difference of the stance-phase and one gait cycle durations, paired *t*-test was conducted (*p* < 0.01). For the time-series kinematic and EMG data, 40 cycles for walking and 50 cycles for running sequences were used to condense the movement features for each leg. The ensemble averages and standard deviations (SD) of 40-cycle walking and 50-cycle running data were input into one-dimensional paired statistical parametric mapping (1D SPM) technique (Pataky et al., 2013) to test the temporal side-to-side differences. This ensemble procedure provided enough statistical power to detect any side-to-side difference during cyclic movement. The alpha level for SPM analysis was adjusted to 0.0017 (=0.01/6) for six component comparisons (Robinson et al., 2014). When the SPM test detected significant side-to-side difference in a certain duration within one gait cycle, the effect size (Cohen’s *d*) averaged over the significant duration was calculated. All SPM analyses were implemented using the open-source spm1d code^1^ in Python 3.6.3.

## Results

Since all trials (= 6) showed consistent features, the results of an illustrative third trial are described below.

### Characteristics of Symptom From Video Observation

**FIGURE 1:**
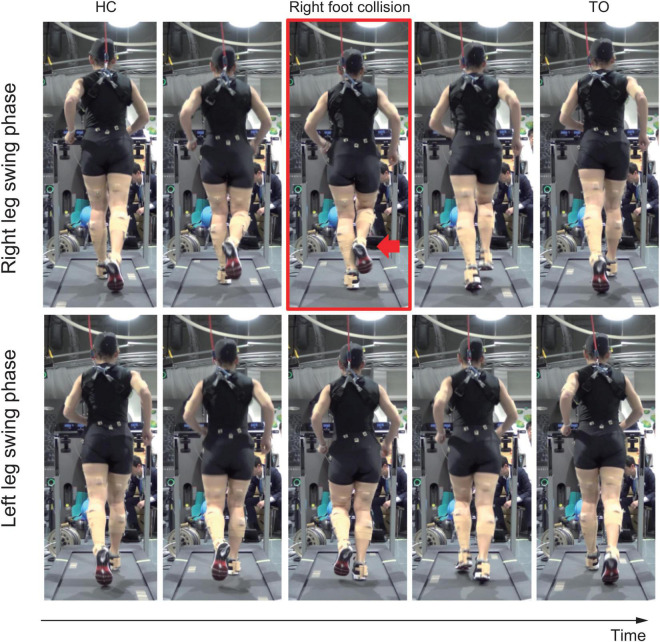
Rear view of the running movement. Upper row shows right leg swing-phase, and lower row shows left leg swing-phase. Red arrow in the red squared panel shows right foot collision with the left calf.

**FIGURE 2:**
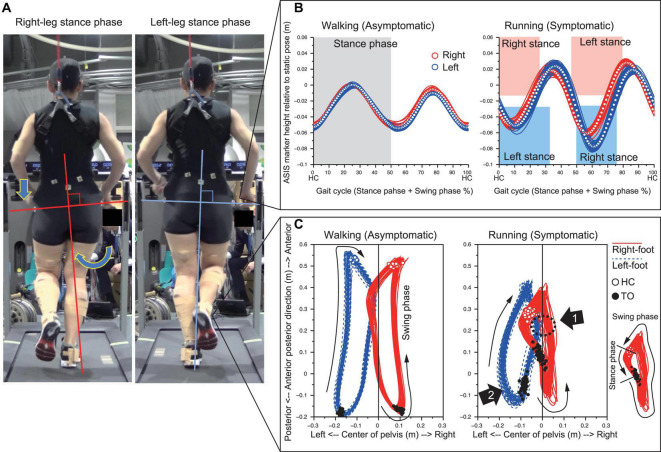
Prominent left pelvis-drop observed during right-leg stance-phase **(A)**. The temporal change of the ASIS markers height illustrated that left pelvis-drop only occurred during right-leg stance-phase of running **(B)**. The top view of 3D trajectory of foot segment with respect to the pelvis coordinate system showed that right-foot trajectory medially shifted and impinged with left-leg area (Arrow 1). The left leg in turn circumducted to escape from right foot interference (Arrow 2) **(C)**.

The medial side of the right foot collided with the calf of the left leg during the right-leg swing-phase in running (Figure 1 and see Supplementary Video). The foot collision consistently occurred during running (53 times collisions in 60 steps), and this phenomenon was seen in only the right foot. The left pelvis-drop in the right-leg stance-phase was significantly greater than that of right pelvis-drop in the left-leg stance-phase (−0.06 (0.003) vs. −0.08 (0.00) m, *p* < 0.05). The large left pelvis-drop induced a medial shift of overall right lower limb segments with respect to the center of pelvis segment (Figures 2A,B). The top view of foot segment’s trajectory with respect to the center line of the pelvis segment illustrated that despite symmetrical foot movement patterns seen during walking, significant side-to-side difference was observed while running. Overall, right foot trajectory was medially shifted and partially impinged with the left foot trajectory at mid-to-late swing-phase (Figure 2C, arrow 1). In contrast, the left foot trajectory was generally shifted laterally, and a prominent circumduction was found in the early swing-phase (Figure 2C, arrow 2).

### Gait Cycle Temporal Asymmetry

**FIGURE 3:**
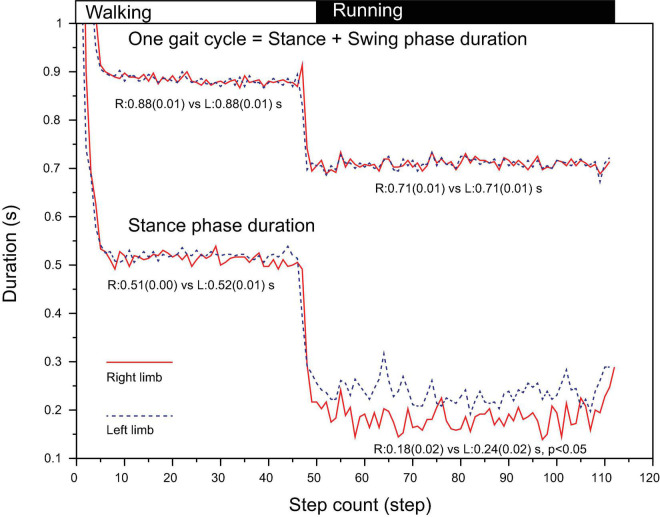
Duration of one gait cycle (HC to HC interval) and stance-phase (HC to TO interval) from an illustrative trial. The stance-phase duration for **right** leg during running was significantly shorter than that of **left** leg. HC, heel contact; TO, toe off.

In walking, the time required for one gait cycle was 0.88s (SD 0.01) for right and 0.88s (SD 0.01) for left leg of which the stance-phase was 0.51s (0.00) for right and 0.52s (0.01) for left leg, showing no statistical significance (Figure 3). In running, although the time taken for one gait cycle did not differ between limbs (0.71s [SD 0.01] vs. 0.71s [SD 0.01], *p* > 0.05), the stance-phase of right leg was significantly shorter than that of left leg (0.18s [SD 0.02] vs. 0.24s [SD 0.02], *p* < 0.05, Figure 3). Due to this phasic difference between limbs, note that both the stance-to-swing transition time and the foot collision time differed between limbs for 100% cycle representation.

### Kinematic Characteristics

**FIGURE 4:**
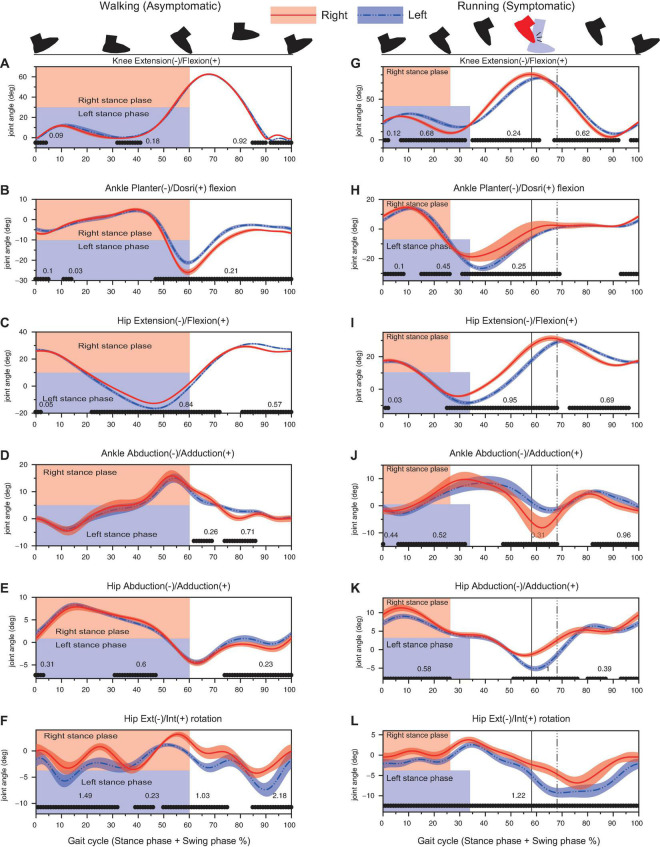
Results of kinematic analysis during walking (left column) and running (right column) assessed by 1D SPM. Black lines with the Cohen’s *d*-value at the horizontal axis of each panel showed significant difference between the right and left legs. Vertical lines denote the timing of foot collision for right leg (solid line) and for left leg (dashed line). Note that the percentage of the stance-phase and the foot collision time was different between the right and left legs in 100% gait cycle representation since the absolute stance-phase duration was significantly different between limbs (as shown in Figure 3).

The side-to-side difference in the time-series kinematic data increased when the athlete started to run (Figure 4). For running data, as the stance-phase for right leg was shorter than that of left leg, the sagittal plane kinematics (knee extension/flexion, ankle plantar/dorsi flexion, and hip extension/flexion) showed phasic differences between limbs, i.e., an early initiation of knee flexion, ankle dorsiflexion, and hip flexion for the right-leg from stance-to-swing transition (30% of gait cycle, Figures 4G–I). The right ankle was additionally abducted by approx. 7° and dorsiflexed by 10° at around the foot-collision phase as compared to the left ankle (Figures 4H,J).

The right hip was further adducted as compared to the left hip during stance-phase (0–30% of gait cycle, Figure 4K). Although the left hip showed a significantly greater abduction around 50–80% of gait cycle (around the time when the foot collided), the right hip did not show a prominent hip abduction (Figure 4K). The cycle-to-cycle variability for hip adduction/abduction angle was relatively small (Figure 4K). The hip rotation angle for right hip was more internally shifted throughout the cycle as compared to the left hip (Figure 4L). The left hip showed a rapid external rotation during 60–70% of gait cycle; however, the right hip did not show such an angular change (Figure 4L).

**FIGURE 5:**
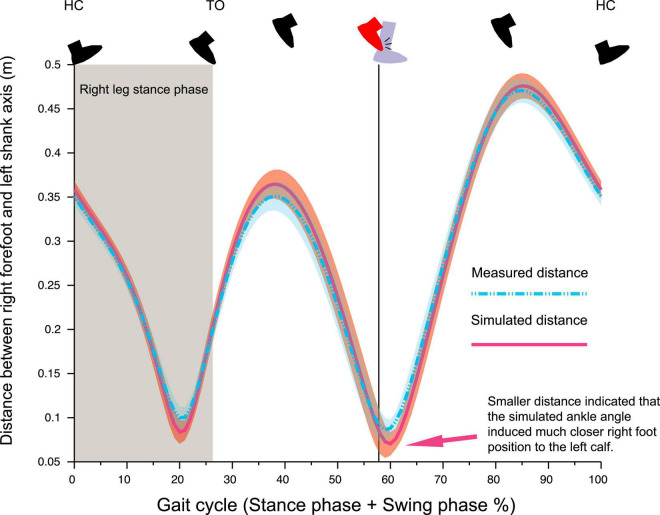
Temporal pattern of the distance between right forefoot (marker FBC) to the left shank segment. If the right ankle was adducted by 7^°^ and plantarflexed by 10^°^ from the observed ankle position as that of left ankle, the distance between right foot and left shank was much closer (Arrow), suggesting the right ankle position observed in this trial was a collision-avoiding strategy.

Results of simulation analysis of foot–calf distance, assuming the absence of 7° abduction and 10° dorsiflexion seen in the right ankle at foot collision phase (Figures 4H,J), indicated that right forefoot would have been about 2.5 cm closer to the left calf (Figure 5).

### Electromyographic Characteristics

**FIGURE 6:**
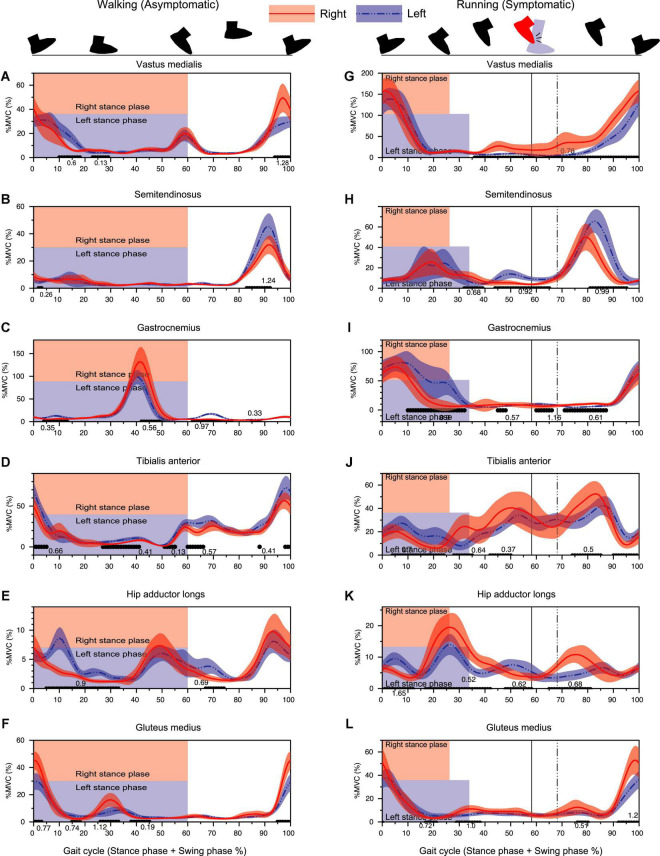
Results of EMG analysis during walking (left column) and running (right column) assessed by 1D-SPM. The black lines with the Cohen’s *d*-value at the horizontal axis of each panel showed significant differences between the right and left legs. Vertical lines denote the timing of foot collision for right leg (solid line) and for left leg (dashed line). Note that the percentage of the stance-phase and the foot collision time was different between the right and left legs in 100% gait cycle representation since the absolute stance-phase duration was significantly different between limbs (Figure 3).

Consistent with the sagittal plane kinematic data, three muscles [vastus medialis (VM), semitendinosus (ST), and gastrocnemius (GC)] contributing to the sagittal plane kinematics showed slight advanced phasic shifts for right leg as compared to the left leg (Figures 6G–I) in running.

For running data, right VM showed a significantly greater activity from 40 to 100% gait cycle with the earlier occurrence pre-activation for subsequent HC at 100% (Figure 6G). Both STs showed a prominent activity at late swing-phase. Left ST showed a significantly greater activity than that of left leg from 80 to 95% of gait cycle (Figure 6H). GC activity initiated slightly before the heel contact (100%) and decreased as the stance-phase finished. Right GC activity was significantly smaller than that of left GC especially at the later part (push-off timing) of stance-phase (Figure 6I).

The right TA showed a significantly greater activity from 30 to 50% of gait cycle as compared to the left TA (Figure 6J). This time duration corresponded to the duration where the less planer-flexed right ankle was observed (Figure 4H).

A prominent increase of hip adductor muscle was observed at the stance-to-swing transition phase (around 20–40% of gait cycle) for both limbs, but the activity for the right hip adductor muscle was significantly greater than that of the left hip. The right hip adductor activation again increased around 65–85% with a significant difference relative to left hip adductor (Figure 6K). Gluteus Medius (GM) activity exhibited a prominent increase toward the heel contact for both limbs. Although right GM activity was significantly greater than that of left GM, right hip showed a significantly greater hip adduction than that of left hip around the HC phase (Figures 6L, 2A,B).

## Discussion

This is the first detailed attempt to quantify the spatiotemporal characteristics of an elite athlete with RD via advanced time-series analysis using motion capture and EMG data. This athlete presented with right-foot collision with the left calf during right-leg swing-phase. However, side-to-side differences were not limited only to the ankle, but was observed throughout the leg. Her lower limb kinematics revealed that there was an asymmetric left pelvic-drop synchronized with an increased right-hip adductor burst, resulting in a medially shifted right-leg trajectory enough to interfere the contralateral left-leg space (Figures 1–4). These findings allowed us to contemplate that the right-foot collision was a secondary phenomenon to abnormal pelvis and hip motor control.

One likely explanation of the foot collision being a secondary phenomenon to the abnormal pelvis and hip control was that the right ankle position (abducted and dorsiflexed than left ankle) prior to the foot collision was a voluntary avoidance strategy rather than an involuntary abnormal movement. Our findings were supported by the results of kinematic simulation analysis which illustrated that the abducted and dorsiflexed right-foot position contributed significantly to widening the distance between the right foot and left leg (Figure 5).

The isolated right TA activity increasing systematically prior to foot collision (30–50% of cycle) without remarkable GC co-activity was suggestive of a non-dystonic type of movement (Figure 6). Prior reports of focal ankle dystonia have shown involuntary co-contraction of agonist–antagonist muscle pair (Ahmad et al., 2018), but in this case, since no such involuntary co-contraction of ankle muscles was observed (Figure 6). The increased right TA activity appearing before foot collision may be an anticipatory muscle activity to configure the dorsiflexed ankle position and widen the distance between the right foot and left calf. Therefore, we believe that the right TA-GC contraction pattern found around foot collision phase also clarifies that the ankle collision was possibly a secondary phenomenon.

Acute, involuntary presentation of symptoms, occurring only during running, and its absence during walking normally, or walking sideways or backward, clinically fit to those with distal ankle dystonia. However, with our current interpretation, we believe this attribute to be a type of segmental dystonia in the truncal and proximal lower limb. In this case, though the symptoms shared several similarities with FTSD at the ankle, the measured kinematic and EMG patterns were quite specific to this athlete. A similar case was reported by Ahmad et al. (2018) in a 56-year-old elite male runner with a 4-year history of involuntary movement in his left limb during running. Some commonalities observed with our case were: (1) an early shift of gait cycle associated with a shorter stance duration in the affected limb and (2) left forefoot scraping the medial aspect of the right ankle. The authors suspected the ankle inversion was due to left foot collision with the right ankle, although motion capture assessment revealed that an excessive hip adduction induced the collision between the distal segments. However, the tonic co-contraction observed between TA and GC by these authors was notably absent within our athlete. Ahmad et al. (2018) also reported a truncal dystonia—a 58-year-old man with 10-year history of long-distance running who exhibited the bilateral posterior pelvic tilt and upward obliquity on the right pelvis, resulting in an abnormal forward and rightward flexion of the trunk (Ahmad et al., 2018). Whereas these cases were comparable to some extent with respect to abnormal truncal or pelvis control, their posture abnormalities were tonic which were considerably different wherein our athlete demonstrated phasic asymmetrical pelvic drop during the right-leg stance-phase. We believe our findings are a worthwhile addition of an uncommon variant of RD symptoms to the knowledge base of task-specific dystonia.

Considering the nature of task-specific dystonia, synchronized visualization of muscle-input (EMG data) and the corresponding movement outcome (3D motion capture data) were vital to elucidate symptom origins in this patient. Additionally, we performed time-series analysis using 1d-spm, aiming to describe slight differences in complex running movement involving cycle-by-cycle movement variation. The ensemble average of 50 running-cycle data input into 1d-spm enabled us to condense the movement features of both affected and unaffected sides, resulting in the identification of statistically meaningful inter-limb difference. Sole visual inspection by experts may not sufficiently quantify the subtle discrepancy between affected and unaffected limbs over whole gait cycle, nor be able to assess muscle activation adequately. Our quantification with motion capture systems with dynamic EMG time-series visualization is therefore beneficial for an accurate understanding of patients’ 3D motion. This will in turn assist expert evaluators to help localize the dystonic origin in the clinical scenarios.

### Limitation

As per literature, the diagnosis of RD should be based on a synthesis of detailed history taking and comprehensive neurological tests, often supported by laboratory data and medical imaging. This study demonstrated the usefulness of additional kinematic and electromyographic assessment. Despite its impact, motion capture system combined with EMG is not necessarily a convenient tool in daily clinical practice because of its significant cost burden in terms of equipment and data analysis. In addition, the measurement method itself is not for diagnosis but merely for biomechanical inference of cause-effect relationships between different muscle elements within the whole-body kinematic chain. Therefore, every effort should be made to increase the practical convenience of such systems.

## Conclusion

This study assessed the kinematic and electromyographic characteristics of a unique RD case. Although the main complaint was that of right foot’s collision with the left leg during the right-leg swing-phase, motion capture assessment suggested that this foot collision may not have originated from the ankle but due to an impaired control mechanism of the right hip and pelvis segment. The multimodal evaluation procedure enabled us to precisely characterize the symptomatology and is therefore a crucial modality for a deeper understanding of the pathogenesis and characteristics of RD.

## Data Availability Statement

The raw data supporting the conclusions of this article will be made available by the authors, without undue reservation.

## Ethics Statement

The studies involving human participants were reviewed and approved by the Osaka University Clinical Research Review Committee. The patients/participants provided their written informed consent to participate in this study.

## Author Contributions

IO, NH, and GR conceptualized and designed the study and wrote the manuscript. NH, HM, and KN provided medical consultations. IO, NH, YU, and TN organized the experiments. IO, NH, YU, YK, and TN performed the experiments and data collection. IO, NH, SK, and GR analyzed the data and performed the statistical analysis. IO, NH, GR, SK, YU, TN, YK, HM, and KN reviewed the manuscript, suggested corrections, and approved its final version. All authors contributed to the article and approved the submitted version.

## Conflict of Interest

The authors declare that the research was conducted in the absence of any commercial or financial relationships that could be construed as a potential conflict of interest.

## Publisher’s Note

All claims expressed in this article are solely those of the authors and do not necessarily represent those of their affiliated organizations, or those of the publisher, the editors and the reviewers. Any product that may be evaluated in this article, or claim that may be made by its manufacturer, is not guaranteed or endorsed by the publisher.

## Footnotes

1
www.spm1d.org
